# Winding-to-ground fault location in power transformer windings using combination of discrete wavelet transform and back-propagation neural network

**DOI:** 10.1038/s41598-022-24434-9

**Published:** 2022-11-23

**Authors:** Pathomthat Chiradeja, Atthapol Ngaopitakkul

**Affiliations:** 1 Faculty of Engineering, Srinakharinwirot University, Bangkok, Thailand; 2 School of Engineering, King Mongkut’s Institute of Technology Ladkrabang, Bangkok, Thailand

**Keywords:** Electrical and electronic engineering, Network models

## Abstract

Power transformers are important equipment in power systems and require a responsive and accurate protection system to ensure system reliability. In this paper, a fault location algorithm for power transformers based on the discrete wavelet transform and back-propagation neural network is presented. The system is modelled on part of Thailand’s transmission and distribution system. The ATP/EMTP software is used to simulate fault signals to validate the proposed algorithm, and the performance is evaluated under various conditions. In addition, various activation functions in the hidden and output layers are compared to select suitable functions for the algorithm. Test results show that the proposed algorithm can correctly locate faults on the transformer winding under different conditions with an average error of less than 0.1%. This result demonstrates the feasibility of implementing the proposed algorithm in actual protection systems for power transformers.

## Introduction

Power transformers, which are essential equipment in power systems, enable the transmission of power between different sections of the network at different voltages. Owing to increased power consumption in the past decade as a result of urbanisation and economic growth, power systems and interconnected power grids have expanded rapidly and become more complex. Power transformers, which play a significant role in these changes, are vulnerable to faults and decrease network reliability. Thus, it is important to establish a protection system to accurately identify faults and isolate them from the system. Locating faults during fault diagnosis can also help operation and maintenance crews respond correctly to faults in power transformers. Winding faults are common in power transformers; they occur inside the transformer and are caused by the degradation of the winding insulation^1,2^. If the fault cannot be addressed quickly and correctly, it can irreparably damage the transformer. Power transformers are typically protected using a combination of overcurrent relays, pressure relays, and differential relays^3^. The Electricity Generating Authority of Thailand (EGAT) currently uses conventional methods to diagnosis faults in power transformers, such as dissolved gas analysis^4^, frequency response analysis (FRA)^5^, and winding impedance measurements^6^. These methods, which have both advantages and disadvantages, can be improved to increase system efficiency and reliability.

Fault analysis has been studied using various techniques to successfully detect, identify, and locate faults in power systems. An interturn fault detection system that uses a primary-side neutral current to indicate fault status has been implemented in power transformers^7^. No-load active power loss and reactive power can be applied to detect interturn faults in power transformers^8^. Using current harmonics as a determining factor, they can correctly detect whether a power transformer is in a fault state^9,10^. A protection system based on the Internet of things can monitor and analyse an obtained signal^11^. For transformer, winding-to-ground fault is an abnormal condition that may lead to damage of the equipment, which is the reason many research has been analysis it characteristic in order to diagnosis it correctly^12,13^. However, implementation is limited because such systems require additional components or are not suitable for specific applications. Thus, mathematical tools have been used to obtain an improved algorithm with broader applicability.

The wavelet transform is a mathematical tool can be used to analyse signals for various purposes. In power system applications, it can overcome the disadvantages of the Fourier transform for signal analysis. The mother wavelet used in the analysis also affects the performance of algorithms, and the performance of various mother wavelets in power system applications has been compared to select the optimal mother wavelet for fault analysis. A previous study illustrated that Daubechies wavelets are suitable for the analysis of transient fault conditions^14,15^. A wavelet-based algorithm for power transformer differential protection correctly detected different types of fault^16^. The continuous wavelet transform has also been implemented by processing the FRA signal to identify winding deformation^17^. Another wavelet-based detection algorithm that requires only the high-frequency components detected earth faults with similar accuracy^18^. The characteristic of the coefficient value also presented in previous work, which demonstrated that this characteristic can be used to for fault diagnosis application^19^. These applications have provided insight into the suitability of wavelets as a mathematical tool to analyse transient fault signals. However, improved accuracy is desirable.

In recent years, artificial intelligence are another tool that has been widely used and able to changing the way interact with the world^20^; methods such as support vector machine^21^, decision tree^22^, and artificial neural networks (ANNs)^9,23,24^ are often employed. Various neural networks provide different suitable objectives and applications. For example, the PNN suitable for is widely used in classification and pattern recognition problems. Convolutional neural network (CNN) is a class of artificial neural network (ANN), most commonly applied to analyze visual imagery. Long short-term memory (LSTM) is an artificial neural network used in the fields of artificial intelligence and deep learning. The application of LSTM for example is handwriting recognition, speech recognition, video games etc. Although there are many types of neural networks, only a few neuron-based structures are commonly used for solving various power system problems. In the case of back-propagation neural network (BPNN), it is one of the first kind of neural networks that are widely researched and applied for fault detection owing to its effectiveness to solve almost all types of problems and do not require a rapid response, such as fault detection, design optimization, and not very short-term forecasting, and its ability to detect, classify, and locate faults in power systems has been demonstrated^25–27^. The success of BPNN is owning to the process of calculating the error and propagating back to the network by an optimizer, which provides very accurate results. From the previous research, we used BPNN to classify internal and external faults in power systems with the power transformer, which can be achieved satisfy accuracy^28^. In addition, the activation functions used in BPNNs provide flexibility for different applications^29^. Its functionalities make it preferable to other neural network types.

A literature review revealed that power transformer faults can significantly affect network operation. Thus, an accurate fault locating algorithm can help operation and maintenance crews address the issue correctly and quickly. However, most studies have focused on the effects of the magnetising inrush current and the discrimination of the magnetising inrush current from internal faults, including fault classification. However, the detection of the locations of winding-to-ground faults is rarely discussed. The ability to identify fault locations along transformer windings would be highly useful. Therefore, this study investigated a method of locating winding-to-ground faults in a two-winding three-phase transformer that requires less complex analysis than conventional methods.

**Figure 1:**
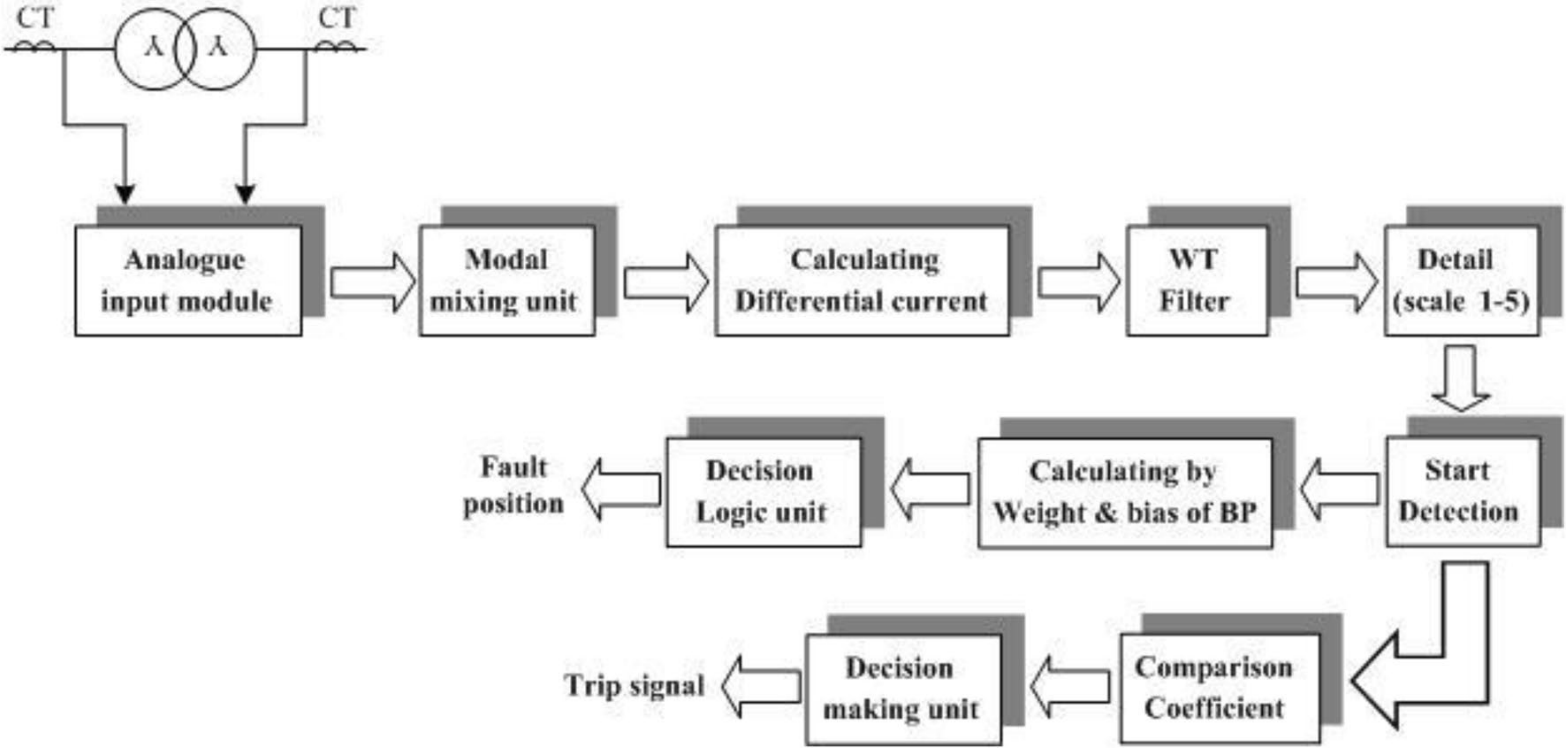
Diagram of power transformer fault detection and location algorithm.

This paper proposes an algorithm for detecting and locating winding-to-ground faults using a wavelet transform and BPNN. To evaluate the performance of the algorithm, the ATP/EMTP software^30^ was used to simulate a power transformer and generate fault signals in a system modelled on an actual EGAT two-winding three-phase transformer in Thailand. Figure 1 shows a simplified flowchart of the proposed algorithm, which can send signals to a trip relay and locate a fault in a power transformer winding. The algorithm uses the current waveform obtained from monitoring units on windings of various sizes and applies a discrete wavelet transform (DWT) to extract the coefficients of the first scale. The comparison coefficient is used as the input for training the neural network. Suitable combinations of activation functions for the hidden layers and output layer of the BPNN are also selected.

This paper is organised as follows. The wavelet transform is explained in detail and the equations are given in "Wavelet transforms" section. The transformer model and parameter calculation are presented in section "Transformer winding model”". The case study used to simulate the fault signal and evaluate the algorithm are described in "Case studies and system characteristic" section. “Neural network decision algorithm and simulation results” section presents the results of the proposed fault location algorithm. Finally, the conclusions of the study are presented in “Conclusions” section.

## Wavelet transforms

The wavelet transform is similar to conventional methods such as the Fourier transform, but it has advantages over the Fourier transform for the analysis of transients resulting from faults in power systems. The reason is that the desired information in this type of application lies in both the frequency and time domains. The DWT has been used to analyse signals obtained from systems. A wavelet is a small localised wave of finite duration with a particular shape and an average value of zero. The wavelet transform is a tool that divides data, functions, or operators into different frequency components and then studies each component with a resolution suitable for its scale^31,32^. The transform enables fine adjustment of the band being analysed so that the high- and low-frequency components are detected accurately. The wavelet transform can expand signals using a shift in time or a translation as well as by compression in time or the dilation of a fixed wavelet function called the mother wavelet. Wavelet transforms in which the analysis is scaled by a factor of two are called DWTs and are written as follows.

1 $$ DWT\left( {m,n} \right) = \frac{1}{{\sqrt {2^{m} } }}\sum\limits_{k} {f\left( k \right)\psi \left[ {\frac{{n - k2^{m} }}{{2^{m} }}} \right]} $$

where $\psi \left[ {\frac{{n - k2^{m} }}{{2^{m} }}} \right]$ is the mother wavelet.

The Daubechies 4 (db4) mother wavelet was selected for the DWT because of its suitability for analysing transients in power systems^14,30–32^.

## Transformer winding model

**Figure 2:**
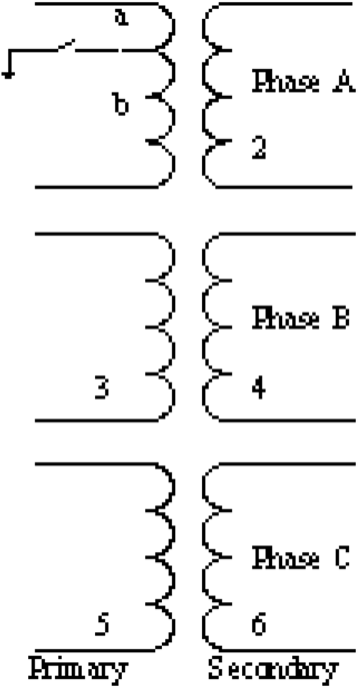
ATP/EMTP model of power transformer with winding-to-ground fault^33^.

**Figure 3:**
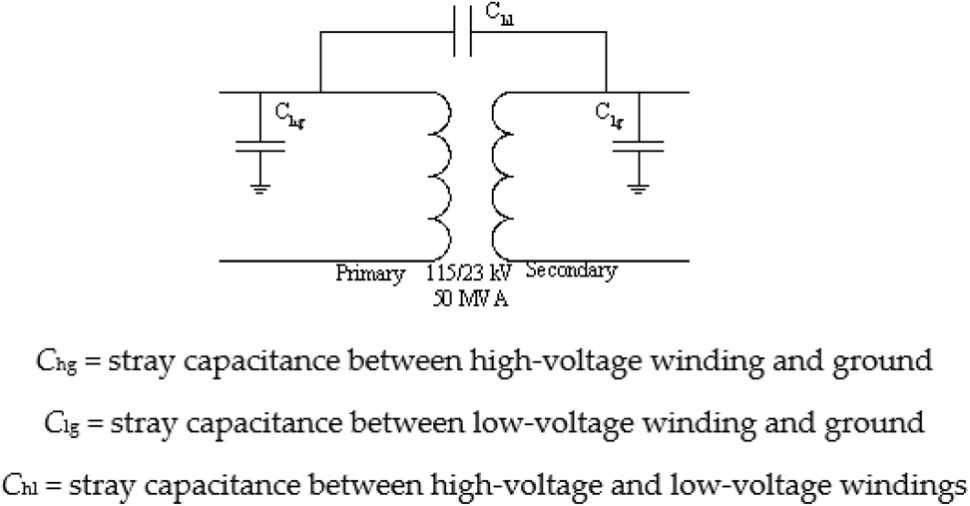
Diagram of two-winding transformer showing the effects of stray capacitances^36^.

A two-winding three-phase transformer with separate primary and secondary windings for each phase was used in this study. An internal fault in the transformer was simulated by modifying the BCTRAN subroutine in the ATP/EMTP software^30^. BCTRAN typically uses a 6 × 6 inductance matrix to represent a transformer; however, because the internal fault condition (specifically, a winding-to-ground fault) is considered here, a 7 × 7 matrix was used^33^. The model was validated and its accuracy was proven by comparison with measurement results. However, the effects of high-frequency components that may appear during faults are not included in this model. The fault types and fault locations have been shown to affect the frequency responses of the transformer^34^. In addition, it has been proved that transient-based protection using the high-frequency components of fault currents can be used to locate and classify faults in transmission lines^35^. Therefore, it is useful to investigate the high-frequency components superimposed on the fault current signals to develop a transient-based protection system for a transformer. Thus, in this study, the transformer model proposed by Bastard et al.^33^ (Fig. 2) is combined with a high-frequency model recommended by an IEEE working group^36^, which includes the capacitances of the transformer (Fig. 3). The model is used to simulate winding-to-ground faults on the transformer windings.

The process for simulating winding-to-ground faults based on the BCTRAN routine of EMTP can be summarised as follows.

First step: Compute matrices R and L of the power transformer from the manufacture test data^37^ without considering winding-to-ground faults^33^, as shown in Eqs. (2) and (3).

2 $$ \left[ R \right] = \left[ {\begin{array}{*{20}c} {R_{1} } & \cdots & 0 \\ \vdots & \ddots & \vdots \\ 0 & \cdots & {R_{6} } \\ \end{array} } \right] $$

3 $$ \left[ L \right] = \left[ {\begin{array}{*{20}c} {L_{1} } & {L_{12} } & \cdots & {L_{16} } \\ {L_{21} } & {L_{2} } & \cdots & {L_{26} } \\ \vdots & \vdots & \ddots & \vdots \\ {L_{61} } & {L_{62} } & \cdots & {L_{6} } \\ \end{array} } \right] $$

Second step: Modify Eqs. (2) and (3) to obtain the new internal winding fault matrices $\left[ R \right]^{*}$ and $\left[ L \right]^{*}$, as shown in Eqs. (4) and (5), respectively^33^.

4 $$ \left[ R \right]^{ * } = \left[ {\begin{array}{*{20}c} {R_{a} } & 0 & 0 & 0 & 0 & 0 & 0 \\ 0 & {R_{b} } & 0 & 0 & 0 & 0 & 0 \\ 0 & 0 & {R_{2} } & 0 & 0 & 0 & 0 \\ 0 & 0 & 0 & {R_{3} } & 0 & 0 & 0 \\ 0 & 0 & 0 & 0 & {R_{4} } & 0 & 0 \\ 0 & 0 & 0 & 0 & 0 & {R_{5} } & 0 \\ 0 & 0 & 0 & 0 & 0 & 0 & {R_{6} } \\ \end{array} } \right] $$

5 $$ \left[ L \right]^{ * } = \left[ {\begin{array}{*{20}c} {L_{a} } & {M_{ab} } & {M_{a2} } & {M_{a3} } & {M_{a4} } & {M_{a5} } & {M_{a6} } \\ {M_{ba} } & {L_{b} } & {M_{b2} } & {M_{b3} } & {M_{b4} } & {M_{b5} } & {M_{b6} } \\ {M_{2a} } & {M_{2b} } & {L_{2} } & {M_{23} } & {M_{24} } & {M_{25} } & {M_{26} } \\ {M_{3a} } & {M_{3b} } & {M_{32} } & {L_{3} } & {M_{34} } & {M_{35} } & {M_{36} } \\ {M_{4a} } & {M_{4b} } & {M_{42} } & {M_{43} } & {L_{4} } & {M_{45} } & {M_{46} } \\ {M_{5a} } & {M_{5b} } & {M_{52} } & {M_{53} } & {M_{54} } & {L_{5} } & {M_{56} } \\ {M_{6a} } & {M_{6b} } & {M_{62} } & {M_{63} } & {M_{64} } & {M_{65} } & {L_{6} } \\ \end{array} } \right] $$

Third step: Simulate the interwinding capacitance and earth capacitance of the high- and low-voltage windings by adding the lumped capacitance connected to the terminals of the transformer.

## Case studies and system characteristic

**Figure 4:**
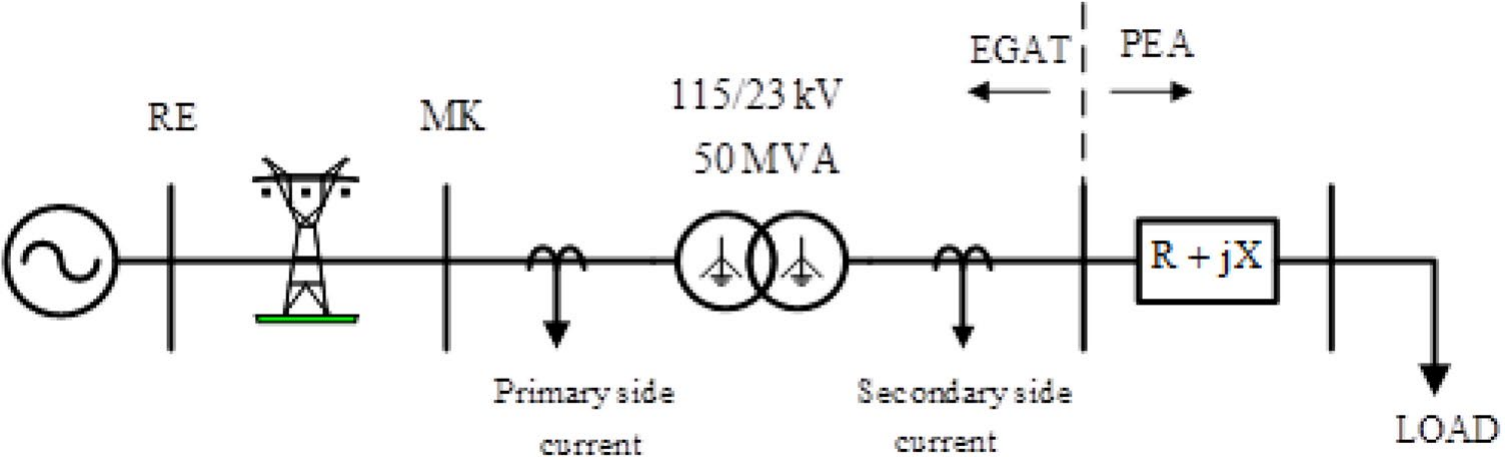
Single-line diagram of transmission line section used in case study.

The ATP/EMTP software^30^ was used to simulate the system signal under normal and fault conditions (sampling rate: 200 kHz, sampling time: 5 μs) The substation used in this case study was modelled after a portion of the 115 kV EGAT transmission system that is connected to a 23 kV Provincial Electricity Authority (PEA) distribution line. The power transformer used in this substation is a two-winding, three-phase step-down transformer (50 MVA, 115/23 kV); the configuration and specifications supplied by the manufacturer^37^ were used. Figure 4 shows a single-line diagram of the power transformer and connected components. In addition, the configuration of the parameter in power transformer that using in the case study can be summarize as shown in Table 1. A thorough simulation was performed to evaluated the proposed algorithm under various conditions. The parameters of the simulations are as follows.

- Primary and secondary windings (high- and low-voltage coils) with various phases in which a fault occurs were used.
- The inception angle of the fault signal (the phase A voltage is used as a reference) was varied from 0° to 330° in steps of 30°.
- The fault location on the winding, measured as the distance from the terminal of the coil, was varied from 10 to 90% in steps of 10%.
- The fault resistance was 5 Ω.

**Table 1:** Configuration of the power transformer using in case study.

Parameter	Value
Rated voltage (Primary/Secondary)	115/23 kV
Rated current (Primary/Secondary)	251.02/1255.11 kA
Connection	Ynyn0
Rated power	50 MVA
Excited loss (Positive/Zero)	22.18/221 kW
Excited current (Positive/Zero)	0.922/200 A
Excited voltage (Positive/Zero)	23/1.993 kV
Short circuit loss (Positive/Zero)	148.10/345 kW
Short circuit current (Positive/Zero)	251.07/200.2 A
Short circuit voltage (Positive/Zero)	14.44/3.224 kV
Stray capacitor (High voltage winding)	12,491.62 pF
Stray capacitor (Low voltage winding)	21,795.15 pF
Stray capacitor (Between high voltage and low voltage winding)	17,424.32 pF

**Figure 5:**
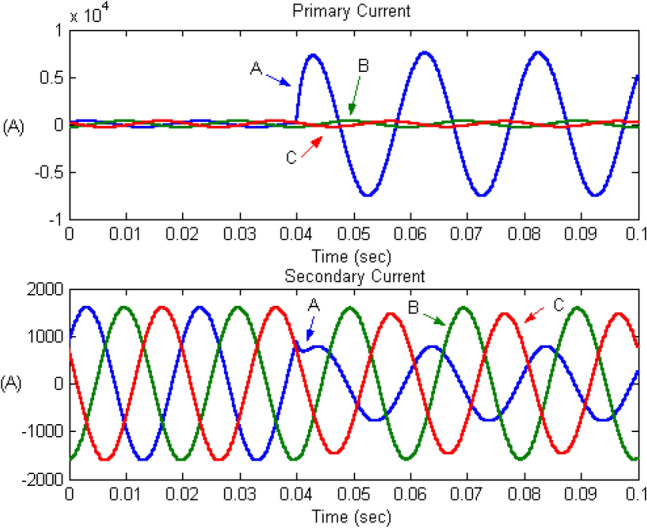
Primary and secondary currents under phase A winding-to-ground fault occurrence on the high-voltage winding at 10% of the length.

**Figure 6:**
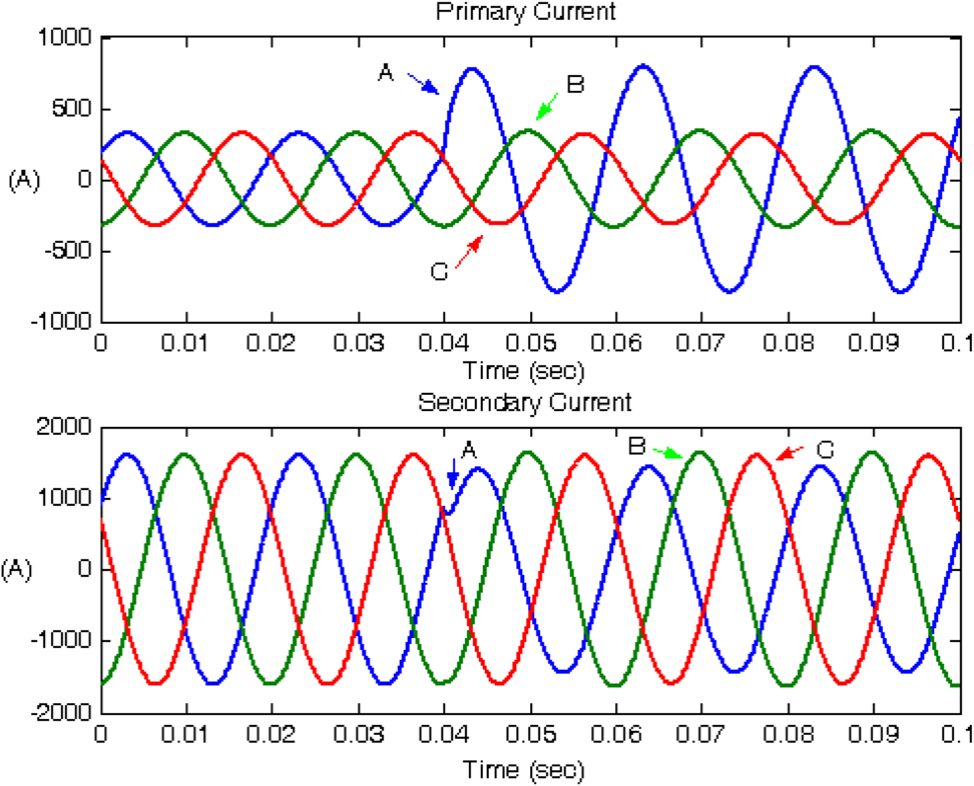
Primary and secondary currents under phase A winding-to-ground fault occurrence on the low-voltage winding at 10% of the length.

Faults in power systems significantly affect the current signal, which thus can be used to determine whether a fault has occurred in the system. Thus, the proposed fault detection algorithm is based on the application of the DWT to the three-phase current waveform obtained from measurement devices on both the primary and secondary sides of the power transformer. Figures 5 and 6 show examples of the three-phase current signal obtained from the primary and secondary sides for a fault from winding phase A to ground in the primary (high-voltage) winding and secondary (low-voltage) winding, respectively, using the ATP/EMTP software^30^.

During the fault, the current of the fault phase on the primary side increased significantly owing to current flow through the fault location. By contrast, the current on the secondary side decreased because less power was transferred owing to the fault on the primary side. However, a fault on the secondary side caused a small transient and a smaller decrease in the current measured from the secondary side. This behaviour can be used to diagnosis the fault status of the system.

**Figure 7:**
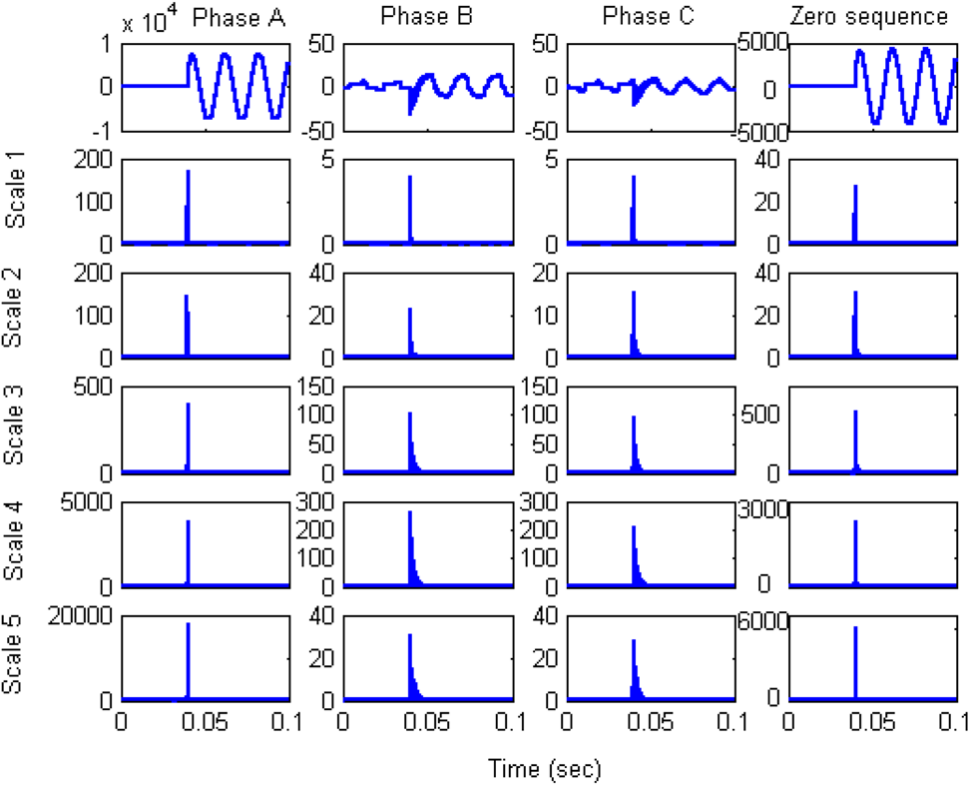
Coefficients of three-phase and zero-sequence differential currents under phase A winding-to-ground fault on high-voltage (primary) winding at 10% of the length.

**Figure 8:**
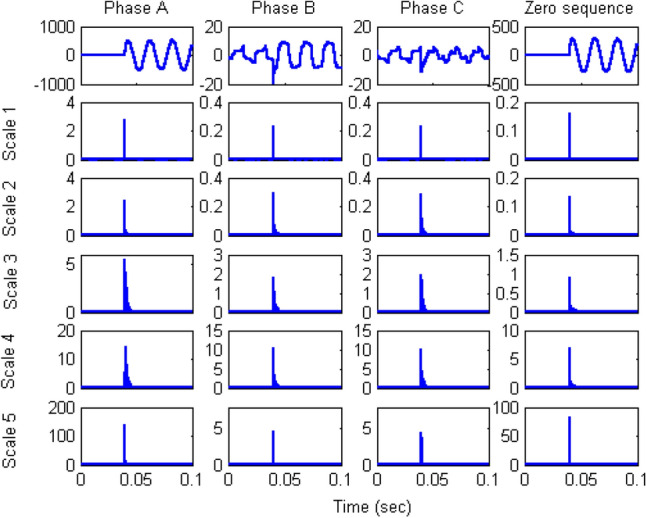
Coefficients of three-phase and zero-sequence differential currents under phase A winding-to-ground fault on low-voltage (secondary) winding at 10% of the length.

The current signals obtained from the primary and secondary sides of the power transformer were used to calculate the differential current. The three-phase current was also transformed into the positive-, negative-, and zero-sequence current. The quarter-cycle of the three-phase current and zero-sequence waveform after fault inception were used as input. The wavelet transform extracts waveform coefficients of scale 1 to scale 5. The coefficient value is extracted from the signal and then squared to emphasized the change in the coefficient value, as shown in Figs. 7 and 8 for faults on the primary and secondary winding, respectively.

The extracted coefficients show a strong peak when a fault occurs in the system and then decrease to the normal level. This behaviour results from the properties of wavelets, which react during transients but not in the steady state. The coefficient output of the wavelet transform was used to construct the decision algorithm by trial and error. The algorithm was applied using coefficients of different scales, and the scale-1 coefficients were found to be sufficiently distinct for the algorithm to identify internal fault inception. Thus, only the scale-1 coefficients obtained from the DWT were used as training data for the neural network to obtain a fast algorithm.

## Neural network decision algorithm and simulation results

**Figure 9:**
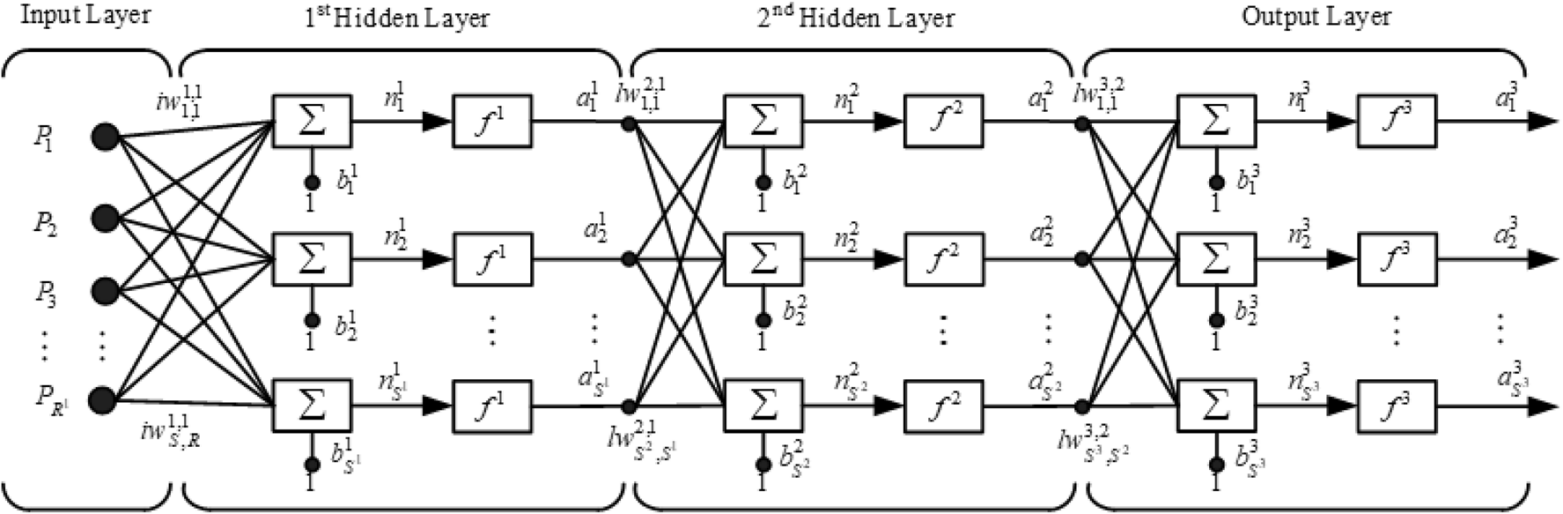
BPNN used in proposed algorithm.

Artificial intelligence, in particular neural networks, has been widely applied in various fields, including electrical engineering. The BPNN is a neuron-based structure that is suitable for pattern recognition and fault classification. It consists of three layers: an input layer, at least one hidden layer, and an output layer. These layers are connected by weights and biases. The proposed algorithm uses a BPNN architecture with one input layer, two hidden layers, and one output layer, as shown in Fig. 9.

Two hidden layers are used in the neural network structure for the algorithm because of the functional advantage over a structure with one hidden layer. However, different activation functions also provide a wide range of accuracy for the algorithm. Thus, suitable activation functions for the fault location algorithm for a power transformer were selected. The hyperbolic tangent sigmoid, logistic sigmoid, and linear functions were considered. Table 2 lists the 12 combinations of activation functions used in the hidden and output layers.

**Table 2:** Combinations of activation functions for training neural networks.

Activation function		
First hidden layer	Second hidden layer	Output layer
Hyperbolic tangent sigmoid	Logistic sigmoid	Linear
		Logistic sigmoid
		Hyperbolic tangent sigmoid
	Hyperbolic tangent sigmoid	Linear
		Logistic sigmoid
		Hyperbolic tangent sigmoid
Logistic sigmoid	Logistic sigmoid	Linear
		Logistic sigmoid
		Hyperbolic tangent sigmoid
	Hyperbolic tangent sigmoid	Linear
		Logistic sigmoid
		Hyperbolic tangent sigmoid

The training process for the BPNN can be divided into three parts, as follows^38^.

- Feedforward input pattern
  The feedforward input pattern propagates data from the input layer to the hidden layer and finally to the output layer. The response from the input pattern can be calculated using Eqs. (6) and (7).
  6 $$ a^{2} = f^{2} \left( {lw^{2,1} *f^{1} \left( {iw^{1,1} *p + b^{1} } \right) + b^{2} } \right) $$
  7 $$ o/p_{ANN} = f^{3} \left( {lw^{3,2} *a^{2} + b^{3} } \right) $$
  where.
  *p* = input vector of BPNN,
  *iw*^1,1^ = weights between input layer and first hidden layer,
  *lw*^2,1^ = weights between first and second hidden layers,
  *lw*^3,2^ = weights between second hidden layer and output layer,
  *b*^1^, *b*^2^ = bias in first and second hidden layers, respectively,
  *b*^3^ = bias in output layer,
  *f*^1^, *f*^2^ = activation function (hyperbolic tangent sigmoid function: tanh),
  *f*^3^ = activation function (linear function).
- Back-propagation of associated error
  The associated error of a BPNN is the error between the neural network and target outputs. It is propagated to all the neurons in the layer below and is also used as a reference for the adjustment of the weights and biases.
- Weight and bias adjustment
  The weights and biases were adjusted using the Levenberg–Marquardt (trainlm) process to match the calculated and target outputs. The mean absolute percentage error (MAPE) index was used to determine the efficiency of the BPNN; it is calculated using Eq. (8).
  8 $$ MAPE = \frac{1}{n}*\sum\limits_{i = 1}^{n} {\left| {\frac{{o/p_{ANNi} - o/p_{TARGETi} }}{{o/p_{TARGETi} }}} \right|} *100\% $$
  where *n* is the number of test sets.

**Figure 10:**
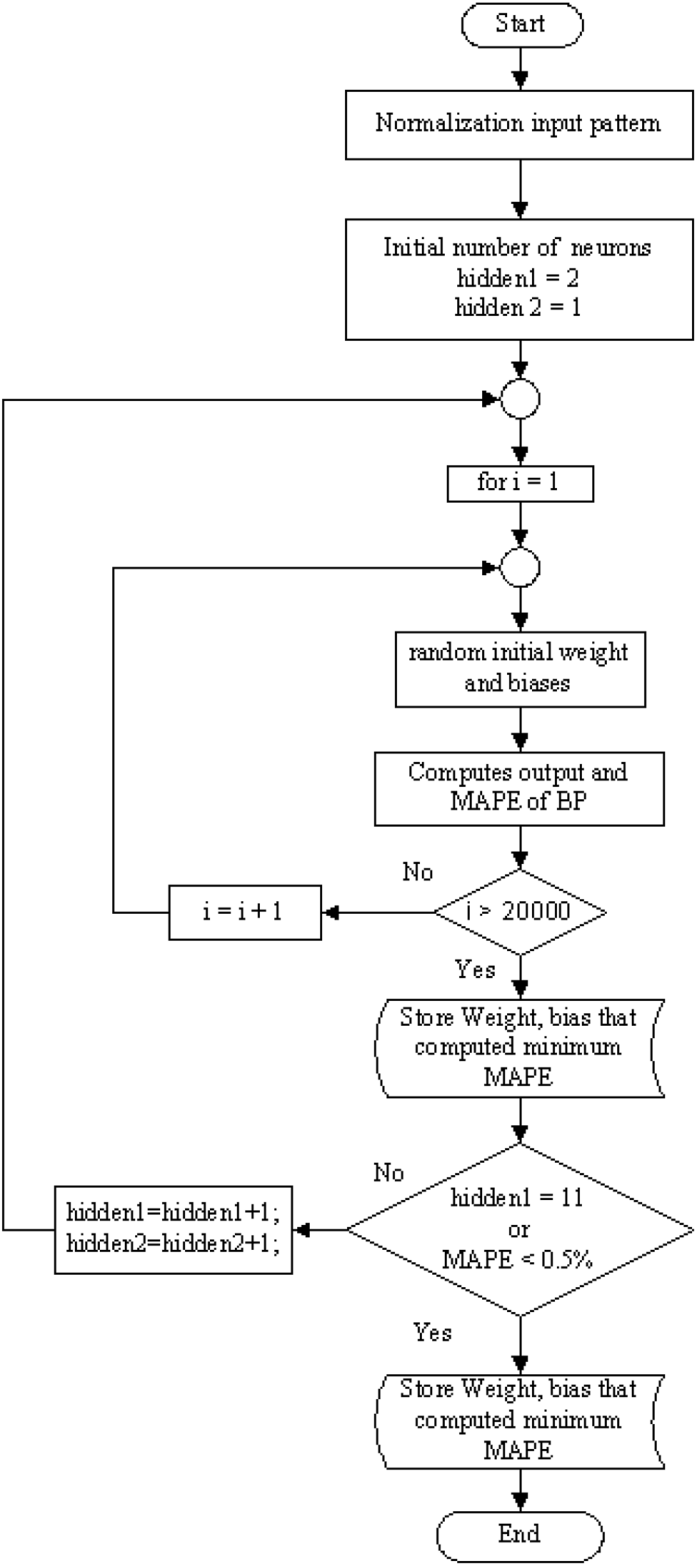
Flowchart of BPNN training process.

The proposed structure of the BPNN based on calculation results consists of four neuron inputs, two hidden layers, and one neuron output. The input pattern of the neural network consists of the scale-1 maximum coefficients (cD1) of the three-phase and zero-sequence currents from the post-fault differential currents. The output variables of the neural network structure are labelled 0.1 to 0.9 depending on the location at which the winding-to-ground fault occurs. The input data sets are normalised and divided into 216 sets for training and 108 sets for testing. To train the neural network, the number of neurons in both hidden layers was increased, and different combinations of activation functions were used to select those with the best performance. The weights and biases were adjusted before the MAPE was computed, and the training cycle was repeated for 20,000 iterations to determine the best MAPE value. Training was stopped when the first hidden layer reached the final number of neurons or the MAPE of the test sets was less than 0.5%. Figure 10 shows a flowchart of the proposed BPNN training process.

**Figure 11:**
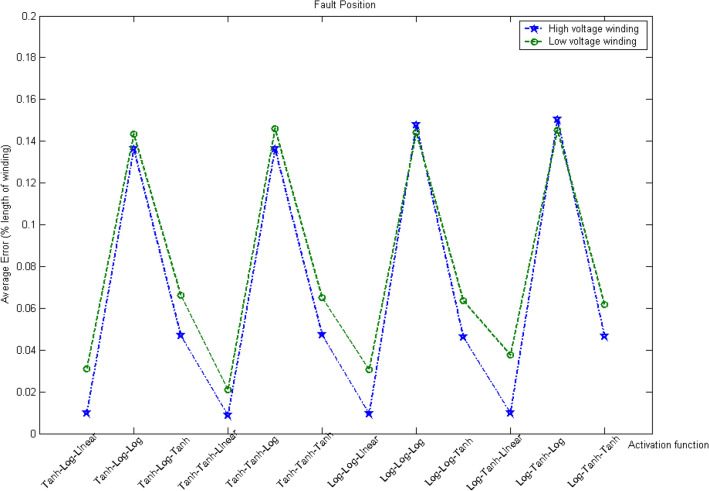
Average errors in fault location for different combinations of activation functions.

After the training process, the BPNN-based decision algorithm was tested using the weight and bias that yielded the minimum MAPE. The stored data were then computed using only the input values, not the target output or calculated values similar to the correct target output. The number of case study and dataset that will be used as an input for the proposed algorithm can be summarize as shown in Table 3. Figure 11 shows the results obtained using various combinations of activation functions and the test set data on both the high- and low-voltage windings.

**Table 3:** Number of case study and data set.

**Number of case study**	
Inception angle (0–330°)	12 cases
Fault location (%Length of Winding)	9 cases
Winding location (Phase/Low and High Winding)	6 cases
Total case study	648 cases
**Number of dataset**	
Training data	216 datasets
Validation data	108 datasets
Unseen data	324 datasets
Total dataset	648 datasets

Four combinations of activation functions were found to provide accurate results with an average error below 5%:

1. Hyperbolic tangent–logistic–linear
2. Hyperbolic tangent–hyperbolic tangent–linear
3. Logistic–logistic–linear
4. Logistic–hyperbolic tangent–linear

After the training process, the algorithm was implemented in the system, and its performance in locating faults was evaluated using the winding-to-ground fault signal. A total of 324 cases under different conditions were examined to verify the algorithm performance and identify suitable combinations of activation functions. Table 4 lists the average error in fault location obtained using the four combinations of activation function that exhibited the best performance.

**Table 4:** Results of test using four best activation function combinations.

Activation function in first hidden layer	Activation function in second hidden layer	Activation function in output layer	High-voltage winding			Low-voltage winding		
			Maximum error	Minimum error	Average error	Maximum error	Minimum error	Maximum error
Hyperbolic tangent sigmoid	Logistic sigmoid	Linear	0.0414	0.0000	0.0099	0.1693	0.0000	0.0309
Hyperbolic tangent sigmoid	Hyperbolic tangent sigmoid	Linear	0.0322	0.0001	0.0089	0.0621	0.0001	0.0211
Logistic sigmoid	Logistic sigmoid	Linear	0.0497	0.0000	0.0094	0.1759	0.0000	0.0307
Logistic sigmoid	Hyperbolic tangent sigmoid	Linear	0.0483	0.0001	0.0098	0.1709	0.0006	0.0377

The four optimal activation function combinations have similar errors for the high-voltage winding. However, for the low-voltage winding, the combination hyperbolic tangent–hyperbolic tangent–linear can achieve higher accuracy with significantly less error than the other combinations.

**Figure 12:**
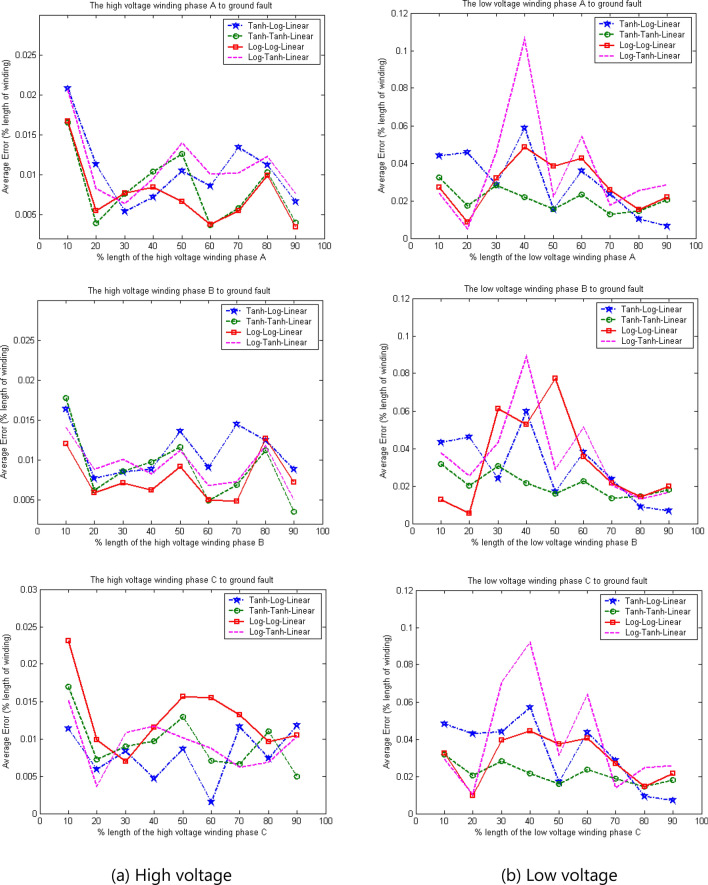
Average error in fault location using four optimal activation function combinations.

Figure 12 shows the average error in fault location for different combinations of activation functions for the high- and low-voltage windings from phase A to phase C. The average error in fault location for the high-voltage winding is 2.5%, whereas that of the low-voltage winding is 6% at various lengths of the transformer winding. These results indicate the performance of each activation function under different conditions. The hyperbolic tangent–hyperbolic tangent–linear combination may not provide the best accuracy under some fault conditions, such as fault locations at 10% and 20% of the length on the low-voltage winding. However, it can provide the best accuracy on average for locating winding-to-ground faults.

**Figure 13:**
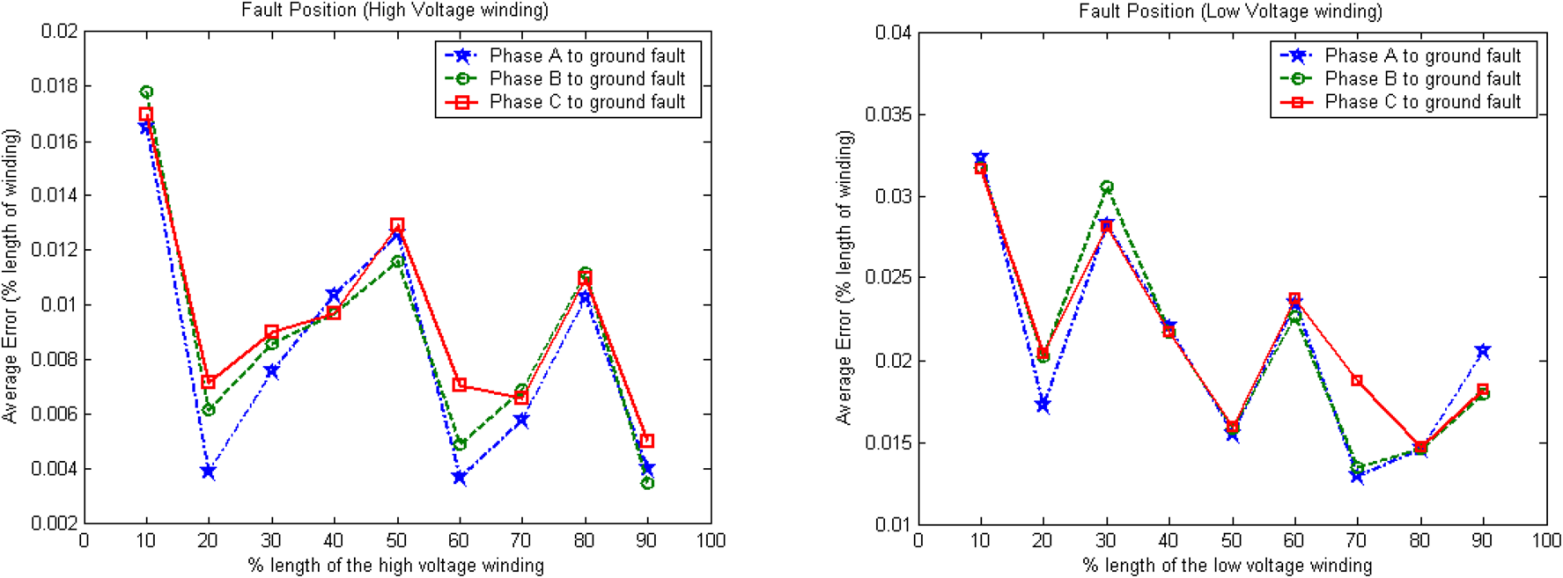
Average error in fault location when algorithm is used with hyperbolic tangent–hyperbolic tangent–linear activation functions.

Table 4 and Fig. 12 show that hyperbolic tangent–hyperbolic tangent–linear is a suitable combination of activation functions. Thus, it was selected as the activation function combination in the BPNN for locating winding-to-ground faults. Figure 13 shows the average error in fault location when the algorithm with these activation functions was applied. For various fault locations on both the high- and low-voltage windings of the three-phase transformer, the accuracy of fault location based on the prediction of the decision algorithm was excellent. The effects of various parameters on the performance of the proposed algorithm are shown in Tables 5, 6, 7, 8.

**Table 5:** Fault location for phase A winding-to-ground faults on high-voltage winding with various inception angles.

Fault type	Inception angle (°)	Actual location (% of length)	Prediction	
			Output	∣Error∣
Winding-to-ground fault, phase A (High-voltage winding)	90	10	0.1125	0.0125
	150	10	0.1243	0.0243
	240	10	0.1121	0.0121
	300	10	0.1169	0.0169

**Table 6:** Fault location for phase A winding-to-ground faults on low-voltage winding with various inception angles.

Fault type	Inception angle (°)	Actual location (% of length)	Prediction	
			Output	∣Error∣
Winding-to-ground fault, phase A (Low-voltage winding)	60	10	0.0886	0.0114
	120	10	0.136	0.036
	210	10	0.1386	0.0386
	330	10	0.0568	0.0432

**Table 7:** Fault location for phase A winding-to-ground faults at various locations on high-voltage winding.

Fault type	Inception angle (°)	Actual location (% of length)	Prediction	
			Output	∣Error∣
Winding-to ground fault, phase A (High-voltage winding)	240	20	0.2005	0.0005
	240	40	0.4054	0.0054
	240	60	0.6042	0.0042
	240	80	0.8025	0.0025

**Table 8:** Fault location results for phase A to ground faults at various locations on low-voltage winding.

Fault type	Inception angle (°)	Actual location (% of length)	Prediction	
			Output	∣Error∣
Winding-to ground fault, phase A (Low-voltage winding)	210	20	0.1943	0.0057
	210	40	0.4265	0.0265
	210	60	0.5871	0.0129
	210	80	0.7968	0.0032

Tables 5 and 6 show the predicted fault location and its error for winding-to-ground faults in phase A on the high- and low-voltage windings, respectively. The actual fault location was fixed at 10% of the length of the winding, and the inception angle of the fault signal was varied. The algorithm predicted the fault location on the high-voltage winding with an error of less than 3% for all inception angles. The prediction for the low-voltage winding was less accurate than that for the high-voltage winding. However, the overall error was below 5%. Thus, the inception angle does not significantly affect the performance of the proposed fault location algorithm.

Tables 7 and 8 list the predicted fault location and its error for winding-to-ground faults in phase A with a fixed inception angle and various fault locations on the high- and low-voltage windings, respectively. The inception angle was fixed at 240° for the high-voltage winding and 210° for the low-voltage winding. The algorithm predicted the fault location with errors of less than 1% for the high-voltage winding. The prediction was again less accurate for the low-voltage winding than for the high-voltage winding. However, the overall error was below 3%. These results demonstrate that the algorithm can locate faults accurately at every possible location.

The application of the proposed fault location method to different winding-to-ground faults in a power transformer revealed that the algorithm can detect faults accurately with errors of less than 0.5%. The fault location, inception angle, and winding voltage do not affect the performance of the algorithm. This study demonstrated that the proposed algorithm can be implemented in power transformer protection systems to provide operation and maintenance crews at electrical utilities with accurate information to respond to faults in power transformers quickly and correctly.

## Conclusions

Power transformers are an essential component in power systems and can be vulnerable to faults, especially internal fault. Thus, a protection system is needed to accurately identify and isolate faults to minimise equipment damage. In addition, the ability to locate faults helps maintenance and operation crews to correctly respond to faults and ensure network reliability. The proposed algorithm uses a combination of the DWT and BPNN to locate winding-to-ground faults in a three-phase power transformer. The scale-1 maximum coefficients of the three-phase post-fault differential current signals and zero-sequence current were used as input to train the BPNN. Case studies of faults under different conditions were used to evaluate the performance of the proposed algorithm. The results demonstrated that the algorithm can detect and locate winding-to-ground faults using the activation function combination hyperbolic tangent–hyperbolic tangent–linear in various layers of the BPNN. The overall accuracy of the proposed algorithm exceeds 95%, and in some cases the accuracy reaches 99.5%. In addition, the fault location performance was not affected by various system parameters. This technique should be useful in identifying and repairing winding-to-ground faults in power transformers. In future work, the algorithm will be improved so that the locations of interturn faults along the windings of the transformer can be identified.

## Data Availability

The datasets used and analysed during the current study available from the corresponding author on reasonable request.
